# Exogenous Ketone Supplementation Enhances the Anti-Epileptic Effect of Levetiracetam in Wistar Albino Glaxo/Rijswijk Rats

**DOI:** 10.3390/nu17101721

**Published:** 2025-05-20

**Authors:** Enikő Rauch, Csilla Ari, Dominic P. D’Agostino, Zsolt Kovács

**Affiliations:** 1 Department of Biology, Berzsenyi Dániel Teacher Training Centre, ELTE Eötvös Loránd University, Károlyi Gáspár tér 4, 9700 Szombathely, Hungary; raucheniko9810@gmail.com (E.R.); kovacs.zsolt@sek.elte.hu (Z.K.); 2 Institute of Biology, University of Pécs, Ifjúság Str. 6, 7624 Pécs, Hungary; 3 Ketone Technologies LLC, Tampa, FL 33612, USA; ddagosti@usf.edu; 4 Behavioral Neuroscience Research Laboratory, Department of Psychology, University of South Florida, Tampa, FL 33620, USA; 5 Laboratory of Metabolic Medicine, Department of Molecular Pharmacology and Physiology, Morsani College of Medicine, University of South Florida, Tampa, FL 33612, USA; 6 Institute for Human and Machine Cognition, Ocala, FL 34471, USA

**Keywords:** levetiracetam, ketone supplement, epilepsy, WAG/Rij rat

## Abstract

**Background:** It has been demonstrated that levetiracetam can decrease absence epileptic activity in both human patients and different types of animal models of absence epilepsy, such as the genetically absence epileptic Wistar Albino Glaxo/Rijswijk (WAG/Rij) rat. It was also suggested previously that exogenous ketone supplements (EKSs)-evoked ketosis not only decreases the number of spike-wave discharges (SWDs) but also enhances the anti-absence epileptic effect of pyrimidine nucleoside uridine in WAG/Rij rats. These findings suggest that EKSs may enhance the efficacy of clinically used anti-epileptic drugs, such as levetiracetam. **Methods:** We investigated the effect of not only levetiracetam (intraperitoneal/i.p. 200 mg/kg) alone and KEKS supplemented food (containing 10% ketone ester/KE and 10% ketone salt/KS in a normal rat chow) alone, but also the combination of levetiracetam and KEKS supplemented food on SWD number and SWD time for 5 days in WAG/Rij rats. For evaluation of SWDs, electroencephalographic (EEG) recordings were performed every day. Moreover, for the measurement of blood glucose and R-beta-hydroxybutyrate (R-βHB) levels, the blood was taken from the tail vein of rats after EEG registration. **Results:** It was demonstrated that the administration of both levetiracetam alone and KEKS food alone decreased the SWD number and time spent in SWD, compared to control. Moreover, after combined administration of levetiracetam with KEKS food, enhanced anti-absence epileptic effect was observed, compared to levetiracetam alone. Blood R-βHB level significantly increased after administration of both KEKS food alone and KEKS food in combination with levetiracetam. Nevertheless, these treatments did not significantly change the blood glucose levels. **Conclusions:** We can conclude that EKSs may be able to enhance the anti-epileptic effect of different drugs, and this combined treatment method may represent a promising new approach and effective therapy against epileptic seizures, especially in treatment-resistant patients.

## 1. Introduction

It has been demonstrated that treatment by the anti-epileptic drug levetiracetam ((S)-2-(2-oxopyrrolidin-1-yl) butanamide) was effective against both focal-onset and generalized-onset seizures not only in several mice and rat epilepsy models, but also in human patients [1,2,3,4]. The anti-epileptic mechanisms of action of levetiracetam are not fully understood. However, the modulatory effect of levetiracetam, among others, through binding to synaptic vesicle protein 2A (SV2A) [3] and, consequently, changes in the vesicular release of neurotransmitters, such as decrease in glutamate release, may be involved in levetiracetam-generated beneficial influences. These effects can decrease excessive excitability in the brain areas involved in epilepsy genesis, resulting in decreased epileptic seizures [1,5,6,7]. Nevertheless, levetiracetam treatment is generally well tolerated and does not cause serious, life-threatening side-effects, but adverse gastrointestinal and central nervous system effects (e.g., vomiting and nausea, as well as somnolence and hyperactivity) were reported [1]. Consequently, a decrease in the clinically applied dose of levetiracetam would be desirable.

Exogenous ketone supplements (EKSs) can induce an increase in the blood level of ketone bodies (nutritional ketosis), such as beta-hydroxybutyrate (βHB) [8,9]. It has been suggested that ketone bodies could be utilized not only for supporting adenosine triphosphate (ATP) production, leading to an increase in energy metabolism in the brain cells, but also as endogenous anti-epileptic agents [10,11]. While studies on murine models and human patients with epilepsy demonstrate that ketosis may be one of the main mechanisms of action by which EKSs can evoke anti-seizure/anti-epileptic effects [9,11], the exact mechanism(s) of the ketosis-generated influences on epileptic activity is largely unknown. However, it was demonstrated that EKS-generated ketosis can mitigate epileptic activity, likely through, among others, glutamatergic, gamma-amino butyric acid (GABA) -ergic and adenosinergic systems [10,11,12,13]. Importantly, appropriate doses of EKSs do not typically cause serious side-effects (mostly mild gastrointestinal problems) [14].

Previous studies showed that levetiracetam can decrease absence epileptic activity in human patients [15] and in different types of animal models of human absence epilepsy, such as Wistar Albino Glaxo/Rijswijk (WAG/Rij) rats [2,16,17,18]. Similarly, it was also revealed that EKS-evoked nutritional ketosis can decrease absence epileptic activity (e.g., number of spike-wave discharges/SWDs) in WAG/Rij rats [9,19]. Moreover, an EKS KSMCT (a mix of ketone salt/KS and medium-chain triglyceride/MCT oil) increased the anti-absence epileptic effect of the endogenous anti-seizure agent pyrimidine nucleoside uridine in WAG/Rij rats [20], suggesting that EKSs could enhance the efficacy of anti-epileptic drugs used in human epilepsy treatment. It has also been demonstrated previously that combined administration of levetiracetam with other anti-seizure drugs may enhance the anti-seizure efficacy safely [21]. In addition, both levetiracetam and EKS-evoked ketosis can decrease absence epileptic activity through similar pathways, such as enhancing extracellular GABA levels and adenosine levels, as well as decreasing glutamate release [6,10,12,13,22], suggesting that combined application of levetiracetam with EKSs can enhance their anti-absence epileptic effect. Thus, in this study, the effect of the combined administration of levetiracetam with an EKS KEKS (mix of ketone ester/KE and ketone salt/KS) on SWDs was investigated in WAG/Rij rats. It has been demonstrated that the genetically absence epileptic WAG/Rij rats spontaneously initiate absence-like epileptic seizures leading to the appearance of SWDs on the electroencephalogram (EEG). It was also revealed that SWDs appear first in the hyperexcitable part of the somatosensory cortex named the cortical focus of absence epilepsy genesis [23]. The purpose of this study was to enhance the anti-absence epileptic effect of levetiracetam. Not only the results above, but also the beneficial effects of both levetiracetam and EKSs on balancing the inhibitory and excitatory processes leading to decrease in hyperexcitability in brain areas involved in absence epilepsy genesis [1,9,19] suggest that combined administration of levetiracetam with KEKS can decrease SWDs more effectively, compared to their effects alone. Consequently, we hypothesized that KEKS could enhance the anti-absence epileptic effect of levetiracetam in WAG/Rij rats.

## 2. Materials and Methods

### 2.1. Animals

Ten-months-old male WAG/Rij rats (*n* = 21; 320–358 g) were used for the experiments. The animals came from the breeding colony at Eötvös Loránd University (Savaria University Centre, Szombathely, Hungary) and were housed 3 or 4 per cage under stable conditions of temperature (at 22 ± 2 °C), 12:12 h light-dark cycle (light on at 08:00 a.m.) and feeding (free access to food and water).

All procedures involving animal care, surgeries and treatments were conducted in conformity with local and international law and polices (Hungarian Act of Animal Care and Experimentation: 1998/XXVIII/243; European Communities Council Directive: 86/609/EEC) under license number VA/ÉBÁF-ÁO/00279-4/2021. After the last treatments, the rats were euthanized by isoflurane (5%) using a vaporizer with induction chamber (Stoelting Co., Wood Dale, IL, USA).

### 2.2. EEG Electrode Implantation

Rats were chronically implanted with cortical stainless steel screw electrodes under isoflurane-air mixture (2.0–2.5%) anesthesia. Screw electrodes were implanted epidurally above the frontal cortex (stereotaxic coordinates: AP 2.0 mm and L 2.1 mm) and parietal cortex (AP −6.5 mm and L 2.1 mm), whereas reference and ground electrodes were placed above the cerebellar cortex [24,25]. Finally, to fix electrodes to the skull bones, Dentacrylate cement (Ivoclar, Schaan, Liechtenstein) was applied and Lidocaine ointment (5%; EGIS, Budapest, Hungary) was used to mitigate post-operative pain.

### 2.3. EEG Recording

Rats were allowed to recover for at least 10 days. After the recovery period, to habituate the rats to the treatment and EEG recording procedures, animals were handled daily and were connected to the recording cables and the electroencephalograph once every day for 3 days before the experiments. EEG was recorded with a NIHON-KOHDEN electroencephalograph (Tokyo, Japan) between 1:00 p.m. and 3:30 p.m. (filtering: below 0.3 Hz and above 150 Hz; sampling rate: 500 Hz). For the analog-to-digital signal conversion, the electroencephalograph was attached to a CED 1401 mkII (Cambridge Electronic Design Ltd., Cambridge, UK) [24]. It has been demonstrated that handling evoked stress can generate alteration in the behavior for about 30 min and therefore modify SWDs [9,23]. Consequently, evaluation of SWDs (in 60 min sections) was accomplished between 30 and 150 min of the recording periods. SWDs contain asymmetric spikes and slow waves (discharge frequency within SWDs: 7–11 Hz; SWD duration 1–30 s) [23]; the SWDs were separated from the EEG recordings manually.

### 2.4. Measuring of Blood Glucose and R-βHB Levels

A ketone monitoring system (Precision Xtra™, Abbott Laboratories, Abbott Park, IL, USA) was used for measurement of blood R-βHB (mmol/L) and glucose (mg/dL) levels. This device only detects R-βHB; consequently, the total blood βHB levels (R-βHB + L-βHB) would be higher than is presented in this study. For the measurement, the blood was taken from the tail vein of rats after EEG registration [8].

### 2.5. Treatment of Rats

Previously, it has been demonstrated that intraperitoneal (i.p.) administration of 200 mg/kg levetiracetam alone sub-chronically (5 days) decreased the SWD number and SWD time in WAG/Rij rats [2]. Moreover, it was also demonstrated that ad libitum feeding of WAG/Rij rats by ketone-supplemented normal rat chow (named KEKS food containing 20% ketone supplements) alone mitigated SWD number from 4–5 days of the treatment parallel with increase in blood R-βHB level in WAG/Rij rats [19,24]. Thus, in the recent study, we used levetiracetam (i.p. 200 mg/kg once/day) in combination with KEKS food for 5 days. The paste-like consistency KEKS food contained powdered standard rodent chow, 10% KE (R,S-1,3-butanediol—acetoacetate diester; % by weight), 10% KS (Na^+^- and Ca^2+^-R/L-βHB salt), 1% saccharine as well as water [19,24]. Fresh KEKS food was mixed every day and was served in a Petri dish placed on the bottom of the rat’s cage.

**Figure 1:**
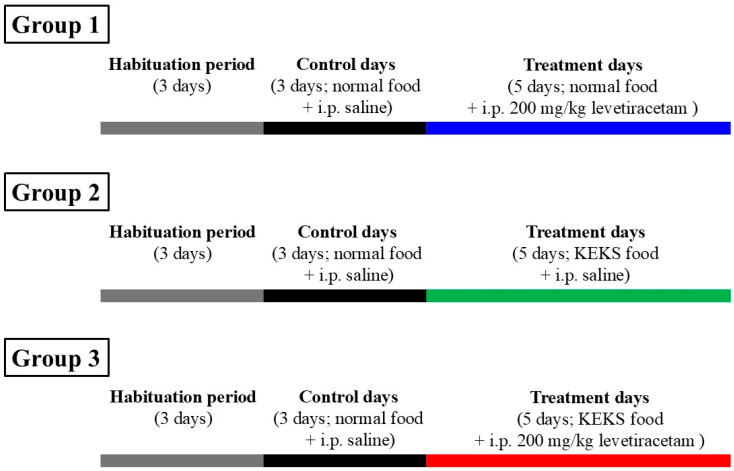
Experimental design. Abbreviations: KEKS food, paste-like KEKS food: powdered standard rodent chow containing 10% KE (R,S-1,3-butanediol—acetoacetate diester; % by weight), 10% KS (Na^+^- and Ca^2+^-βHB salt), 1% saccharine and water; normal food, paste-like normal rat food: powdered standard rodent chow, 1% saccharine and water.

After habituation, experiments were performed on 3 animal groups. The random allocation of animals to the experimental groups was performed by a computer-based random order generator. To determine average control SWD number and SWD duration, all rats were fed by paste-like normal rat food (without ketone supplementation) and received i.p. saline injection (0.3 mL/100 g body weight) on 3 consecutive days (three-day control period) (Figure 1). After the control period, the first group of animals (*n* = 7) was fed by paste-like normal rat food (without ketone supplementation), whereas animals in the second (*n* = 7) and third (*n* = 7) group received KEKS food for 5 days. Moreover, the first and third groups of animals were injected by i.p. 200 mg/kg levetiracetam dissolved in saline (0.3 mL/100 g), whereas animals in the second group were injected by saline (0.3 mL/100 g) for 5 days. After all control and treatment days, EEGs were recorded.

Blood glucose and R-βHB levels were measured on the last control days (control levels), as well as the first and last day of KEKS food administration (second and third animal group). The body weight of the animals in the same groups (second and third group) were measured before KEKS food administration (on the last control days: control levels) and after the last administration of KEKS food.

Animals or data points were not excluded from the experiments and data analysis, and predefined criteria were not used. EEG analysis and statistical evaluation of all collected data were performed by blinded investigators. After all data were collected and analyzed, deblinding was performed.

### 2.6. Statistical Analysis

SWD number, SWD duration, time spent in SWD, body weight of animals, as well as blood glucose and R-βHB levels were presented as means ± standard error of the mean (S.E.M.). The pretreatment control SWD number, SWD duration and time spent in SWD were the grand average determined from the results of control days. For blood levels of R-βHB and glucose, as well as body weight, the control values were counted from the results measured on the last control days. A two-way ANOVA with Šídák’s multiple comparisons test and Tukey’s multiple comparisons test and *t*-test were used to assess results (software: GraphPad Prism version 10.4) [24]. To assess differences across groups and time points, we applied two-way ANOVA followed by either Šídák’s or Tukey’s post hoc multiple comparisons tests using GraphPad Prism (version 10.4). These post hoc tests were chosen specifically to correct for multiple comparisons and control the familywise Type I error rate. Šídák’s test was used for planned pairwise comparisons with fewer comparisons, whereas Tukey’s test was used when all pairwise comparisons were of interest. These methods adjust the *p*-values appropriately to minimize false positive results and ensure the reliability of statistically significant findings. A priori sample size calculation was not performed. However, a post hoc power analysis was conducted using G*Power (version 3.1.9.7) to estimate the achieved statistical power for detecting differences in blood glucose levels between control and treatment groups.

## 3. Results

### 3.1. Effect of Levetiracetam and KEKS Food Alone on SWDs

**Figure 2:**
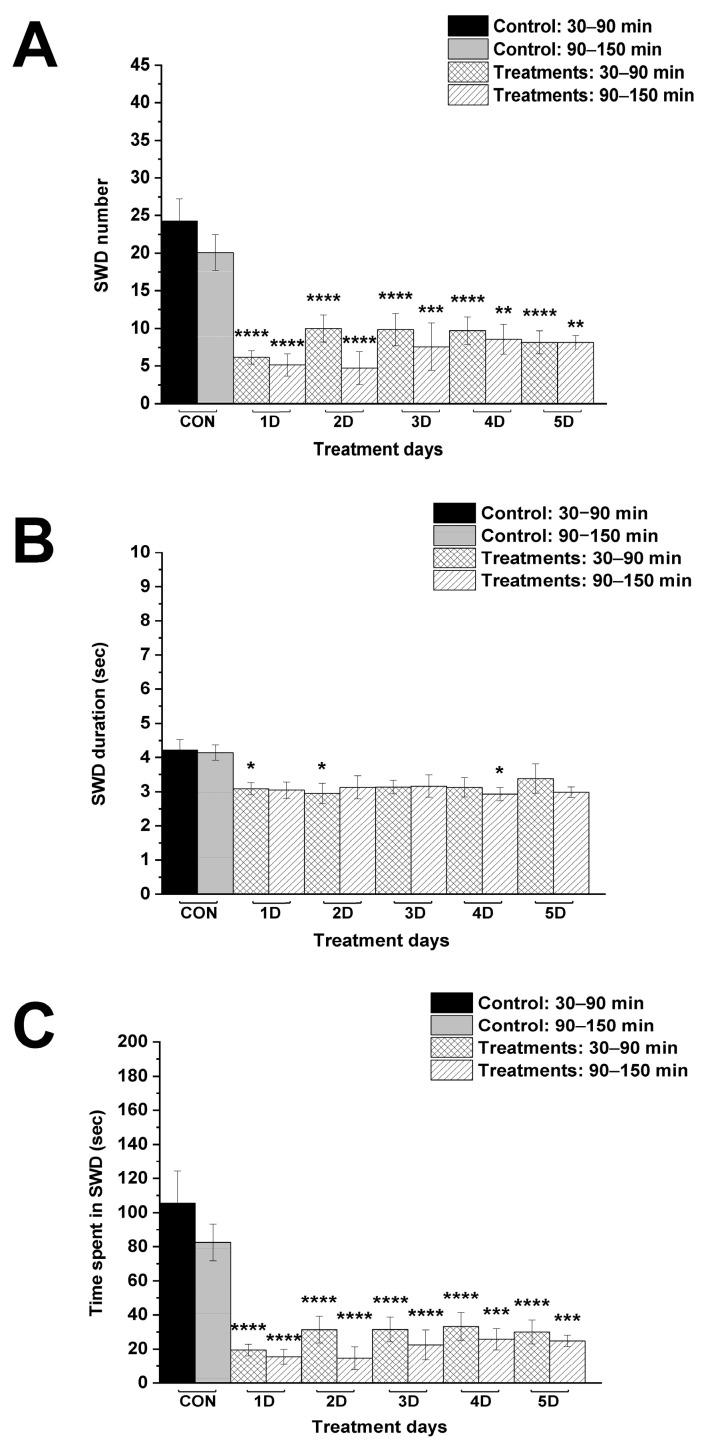
Effect of levetiracetam treatment (i.p. 200 mg/kg) alone on SWD number (**A**), SWD duration (**B**) and time spent in SWD (**C**). Abbreviations: 1D, first treatment day; 2D, second treatment day and so on; CON, control; SWD, spike-wave discharge; level of significance: *, *p* < 0.05; **, *p* < 0.01; ***, *p* < 0.001; ****, *p* < 0.0001.

The i.p. 200 mg/kg levetiracetam alone significantly decreased the SWD number between the first and fifth days of the treatment from 30 to 150 min, compared to control (Figure 2A; Table 1). Time spent in SWD was also decreased, similarly to the change in SWD number after levetiracetam treatment (Figure 2C; Table 1), whereas SWD time significantly decreased only the first (between 30–90 min), second (30–90 min) and fourth (90–150 min) day of the treatment, compared to control (Figure 2B; Table 1).

**Figure 3:**
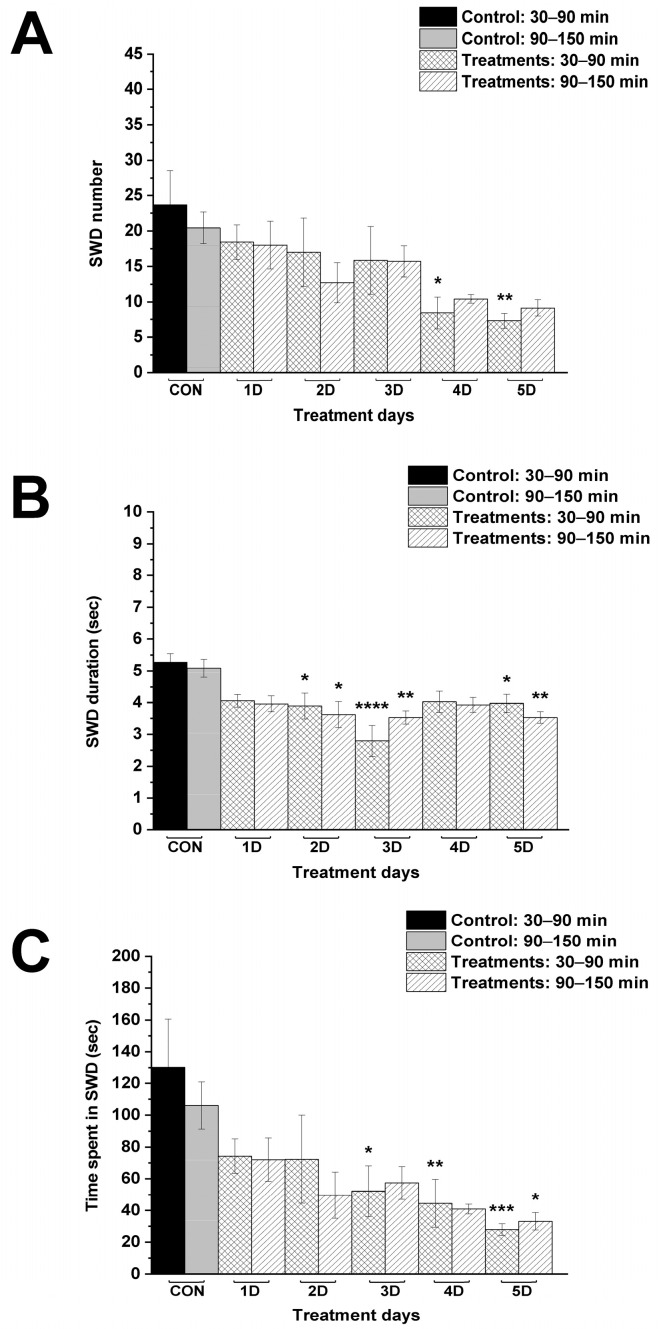
Effect of KEKS food alone on SWD number (**A**), SWD duration (**B**) and time spent in SWD (**C**). Abbreviations: 1D, first treatment day; 2D, second treatment day and so on; CON, control; SWD, spike-wave discharge; level of significance: *, *p* < 0.05; **, *p* < 0.01; ***, *p* < 0.001; ****, *p* < 0.0001.

KEKS food alone gradually reduced the number of SWDs over time, but significant decrease was measured only on the fourth and fifth day between 30–90 min, compared to control (Figure 3A; Table 2). A significant decrease in SWD duration was demonstrated on the second, third and fifth day of KEKS food application between 30 and 150 min (Figure 3B; Table 2). Moreover, time spent in SWD was decreased on the third (30–90 min), fourth (30–90 min) and fifth (30–150 min) day of the treatment (Figure 3C; Table 2).

### 3.2. Effect of Combined Administration of Levetiracetam with KEKS Food on SWDs

**Figure 4:**
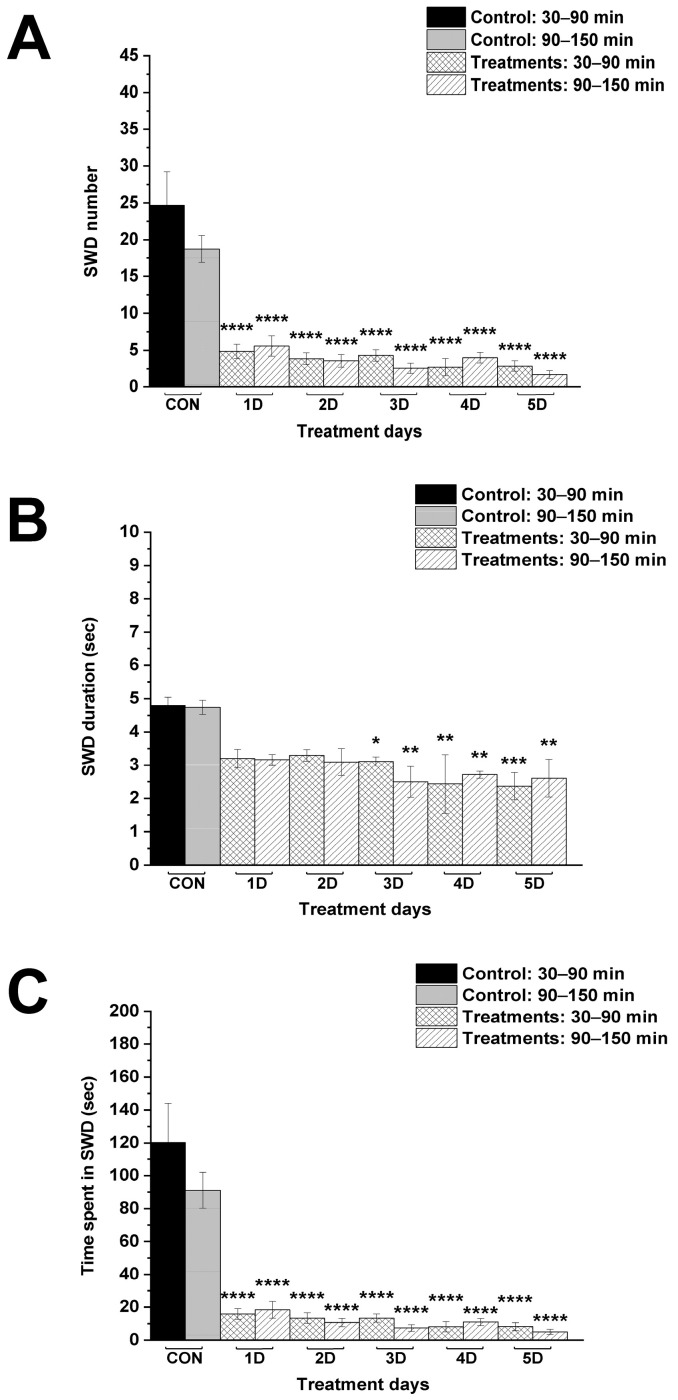
Influence of combined administration of levetiracetam (i.p. 200 mg/kg) with KEKS food on SWD number (**A**), SWD duration (**B**) and time spent in SWD (**C**). Abbreviations: 1D, first treatment day; 2D, second treatment day and so on; CON, control; SWD, spike-wave discharge; level of significance: *, *p* < 0.05; **, *p* < 0.01; ***, *p* < 0.001; ****, *p* < 0.0001.

Combined administration of i.p. 200 mg/kg levetiracetam with KEKS food significantly decreased not only the SWD number and time spent in SWD on every day between 30–150 min (Figure 4A,C; Table 3), but also the SWD duration on the third, fourth and fifth day between 30–150 min, compared to control (Figure 4B; Table 3).

**Figure 5:**
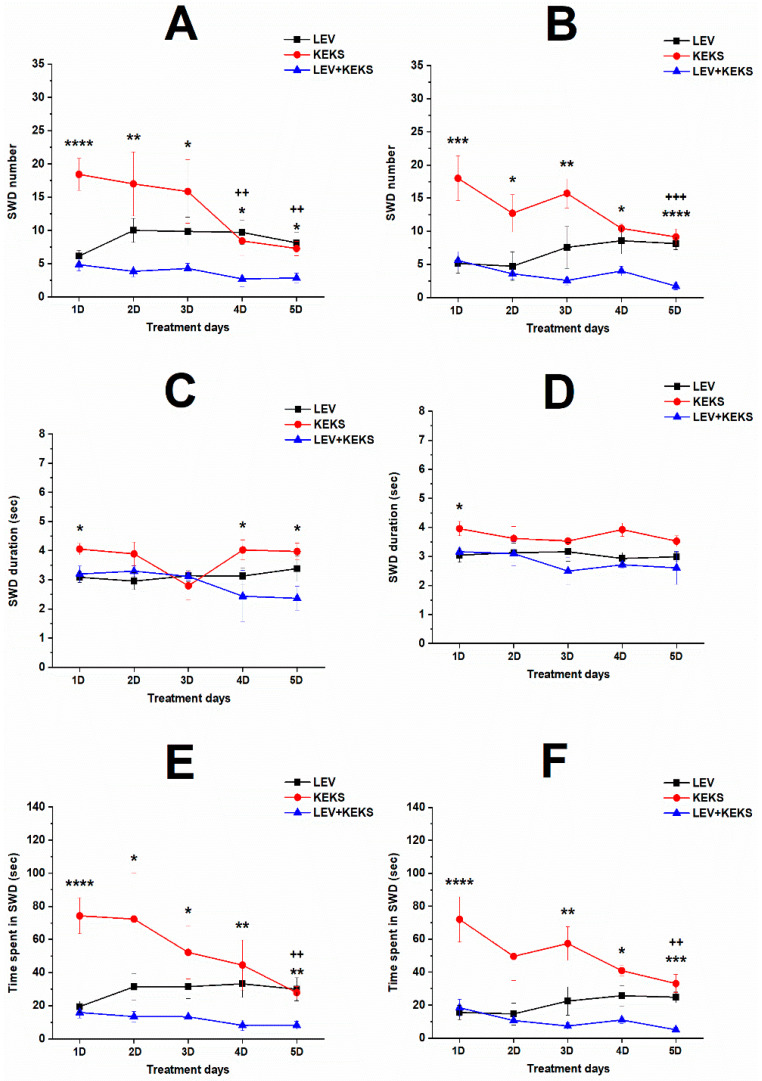
Comparison of influence of levetiracetam treatment (i.p. 200 mg/kg) alone (LEV, black) and KEKS food alone (KEKS, red) on SWD number (**A**,**B**), SWD duration (**C**,**D**) and time spent in SWD (**E**,**F**) with effect of combined administration of levetiracetam with KEKS food (LEV + KEKS, blue) between 30 and 90 min (**A**,**C**,**E**) as well as between 90 and 150 min (**B**,**D**,**F**) after i.p. injection. Abbreviations: 1D, first treatment day; 2D, second treatment day and so on; SWD, spike-wave discharge; level of significance for KEKS food alone vs. combined administration of levetiracetam with KEKS food: *, *p* < 0.05; **, *p* < 0.01; ***, *p* < 0.001; ****, *p* < 0.0001; level of significance for levetiracetam treatment alone vs. combined administration of levetiracetam with KEKS food: ^++^, *p* < 0.01; ^+++^, *p* < 0.001.

The combined administration of levetiracetam with KEKS food significantly and more effectively decreased the SWD number and time spent in SWD on every treatment day (from the first to fifth day), compared to KEKS food-evoked effects (Figure 5A,B,E,F). Nevertheless, the combined administration of levetiracetam and KEKS food decreased the SWD number and time spent in SWD, compared to levetiracetam alone only on the fourth and/or fifth treatment days, when the beneficial effect of KEKS food alone on SWDs was the highest (Figure 5A,B,E,F). It was also demonstrated that combined treatment by levetiracetam and KEKS food decreased the SWD time mainly between 30–90 min (on the first, fourth and fifth treatment days), compared to KEKS food alone (Figure 5C,D). However, combined application of levetiracetam with KEKS food did not enhance the effect of levetiracetam alone on SWD duration (Figure 5C,D).

### 3.3. Changes in Blood R-βHB and Glucose Levels and Body Weight

**Figure 6:**
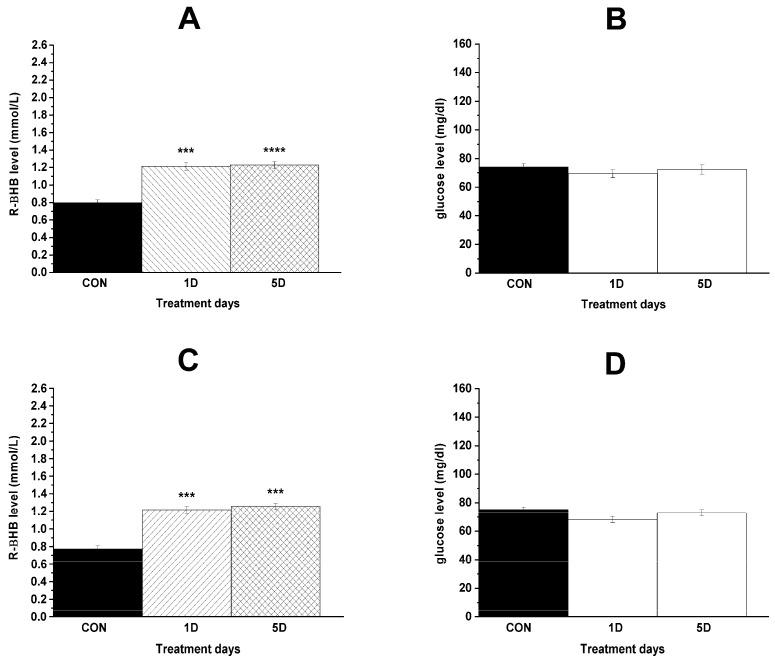
Effect of KEKS food alone (**A**,**B**) and combined administration of levetiracetam (i.p. 200 mg/kg) with KEKS food (**C**,**D**) on blood R-βHB and glucose levels. Abbreviations: 1D, first treatment day; 5D, fifth treatment day; CON, control; R-βHB, R-beta-hydroxybutyrate; level of significance: ***, *p* < 0.001; ****, *p* < 0.0001.

Level of blood R-βHB significantly increased after administration of both KEKS food alone and KEKS food in combination with i.p. 200 mg/kg levetiracetam (Figure 6A,C; Table 4), whereas these treatments did not significantly change the blood glucose levels (Figure 6B,D; Table 4), compared to control. Nevertheless, in relation to blood glucose levels, based on the observed effect size (Cohen’s d = 0.06), sample size (n = 7 per group), and α = 0.05, the post hoc power was calculated to be 0.06.

There was no difference in the body weight of rats after administration of KEKS food alone or i.p. 200 mg/kg levetiracetam with KEKS food, compared to control (body weight of animals on the last control day/on the last day of administration of KEKS food alone or i.p. 200 mg/kg levetiracetam with KEKS food, g ± S.E.M.; control/KEKS food alone: 335.1 ± 4.24/334.9 ± 3.46, *p* = 0.9019; control/combined treatment by levetiracetam and KEKS food: 337.3 ± 4.51/336.3 ± 3.72, *p* = 0.6425).

## 4. Discussion

In this study we demonstrated that administration of both levetiracetam (i.p. 200 mg/kg) alone and KEKS food alone for 5 days can decrease SWD number and SWD time in WAG/Rij rats [2,19] (Figure 2 and Figure 3). Moreover, the current results are the first to demonstrate that ketone supplementation (KEKS supplemented food) in combination with levetiracetam treatment can significantly enhance the levetiracetam-generated beneficial influence on SWD number and time spent in SWD in WAG/Rij rats (Figure 4 and Figure 5).

It has been suggested that modulation of integral membrane protein SV2A may be the primary mechanism by which levetiracetam can decrease epileptic activity [1]. Earlier studies show that a single oral dose of levetiracetam (6.5 mg/kg) and i.p. 80 mg/kg levetiracetam can enter the brain within a quarter of an hour [26,27]. Levetiracetam can diffuse through the blood–brain barrier, brain parenchyma and neuronal membrane and may enter synaptic vesicles reaching and binding to SV2A molecules intravesicularly [26,28], leading to blocking some role of SV2A. For example, levetiracetam inhibits SV2A functions in vesicular priming and exocytosis, therefore normal neurotransmission, which reduces the size of the readily releasable pool of vesicles and synaptic transmission (e.g., release of vesicles containing glutamate), resulting in an anti-epileptic effect [1,5,28]. Moreover, it was also suggested that levetiracetam may enhance SV2A functions resulting in an increase in the presynaptic release of vesicles containing, for example, GABA [4], and, consequently, decrease in epileptic activity [3]. Levetiracetam’s modulation of SV2A may help restore the balance between excitatory and inhibitory neurotransmission, such as the glutamatergic and GABAergic systems, respectively, through alteration of the presynaptic release of glutamate (reducing) and GABA (increasing), leading to decrease in both hyperexcitability and, therefore, epileptic seizures [1,4,6,7].

Levetiracetam likely reduces epileptic activity through multiple pharmacological mechanisms, such as modulation of several calcium and potassium channels, GABAergic, glutamatergic and adenosinergic systems, as well as the inflammatory system. Levetiracetam inhibited the high-voltage activated calcium channels, such as *P*/*Q*- and *N*-type calcium channels in, for example, rat hippocampal neurons [29,30], the influence of which can contribute to the anti-epileptic action of levetiracetam through decrease in both the presynaptic release of neurotransmitters and neuronal excitability. Indeed, levetiracetam inhibited glutamate release through modulation of presynaptic *P*/*Q*-type calcium channel activity in granule cells of the rat dentate gyrus [31], leading to decrease in neuronal excitability. The anti-epileptic effect of levetiracetam may also be related to potassium channel activation, therefore hyperpolarization of neuronal membranes [32] and a moderate reduction in the delayed rectifier potassium currents in neurons, resulting in decrease in repetitive action potential generation [33].

In relation to the effect of levetiracetam treatment through the GABAergic system, the results are very controversial. For example, single administration of levetiracetam (i.p. 170 mg/kg) increased GABA degrading enzyme GABA aminotransferase activity in several rat brain areas, such as the cortex, striatum, hypothalamus and thalamus. Nevertheless, decreased and increased activity of the GABA synthesizing enzyme glutamic acid decarboxylase in the rat striatum and hypothalamus was measured, respectively [34]. Moreover, similar levetiracetam treatment (i.p. 170 mg/kg) markedly decreased the GABA level in the rat cortex and hippocampus [34]. However, chronic levetiracetam administration (250 mg, twice daily for 12 weeks) decreased the GABA level in the posterior cingulate cortex, but the GABA level was not changed in the anterior cingulate cortex/medial prefrontal cortex in human patients [35]. It was also demonstrated that chronic levetiracetam administration (i.p. 54 mg/kg for two weeks) can result in decreased glutamate levels, enhanced GABA synthesis, reversed GABA transport and increase in both the extracellular GABA level and GABAergic inhibition, therefore mitigating epileptic seizure activity [6]. Moreover, levetiracetam (i.p. 200 mg/kg for 5 days) can decrease epileptic activity, likely through increased astrocytic expression of GABA transporter GAT-2/3, reversed GABA transport and, consequently, increased extracellular GABA level-evoked increase in tonic inhibition by extrasynaptic GABA receptors [2]. It has been demonstrated that levetiracetam administration inhibits basal glutamate release [7], the high potassium-induced increase in glutamate overflow [6] and pentylenetetrazole-induced increase in glutamate level in the rat hippocampus [5], as well as glutamate release evoked by potassium-generated stimulation in the rat median prefrontal cortex [36]. It has also been suggested that the application of levetiracetam can modulate the function of alpha-amino-3-hydroxy-5-methylisoxazole-4-propionic acid (AMPA) receptors because levetiracetam decreased the AMPA-induced currents and decreased both the amplitude and frequency of miniature excitatory postsynaptic currents in cortical neurons [37]. Based on these results above, levetiracetam treatment can mitigate epileptic activity through modulation of the GABAergic system and glutamatergic system and therefore decrease in hyperexcitability.

It was demonstrated that levetiracetam may also exert its effect through inhibitory adenosine A_1_ receptors (A_1_Rs), directly or indirectly [38], the influence of which, theoretically, may have a role in the levetiracetam-generated anti-epileptic effect. Indeed, 1,3-dipropyl-8-cyclopentylxanthine (DPCPX) and caffeine decreased the anti-seizure effect of levetiracetam (i.p. 200 mg/kg) in an acute pentylenetetrazole-induced seizure model in mice [22]. Moreover, levetiracetam treatment (i.p. 200 mg/kg for two weeks) increased A_1_R and potassium inwardly rectifying channel 3.2 (Kir3.2) expression whereas it decreased the expression of equilibrative nucleoside transporter ENT1 in the hippocampus and cortex in a pentylenetetrazole-induced chronic kindling mice model. These results suggest that levetiracetam may generate an anti-epileptic effect through the adenosinergic system by means of an enhanced extracellular level of adenosine (because the function of ENT1 is decreased), increased A_1_R activity and A_1_R-evoked increase in activity of Kir3.2, therefore mitigating hyperpolarization of the neuronal membrane in the brain areas [22]. Jamal et al. also demonstrated that levetiracetam can bind to the orthosteric binding site of A_1_Rs and ENTs [22]. Thus, these results strongly suggest that modulation of functions of the adenosinergic system, likely through increase in both the extracellular adenosine level and A_1_R activity, may be one of the most important putative mechanisms of action, by which levetiracetam exerts an anti-epileptic effect.

A substantial number of scientific results have demonstrated that both acute and (sub)chronic application of levetiracetam can generate an anti-seizure effect through neuroprotective and anti-inflammatory influences. For example, levetiracetam treatment (i.p. 40 mg/kg) decreased the kindling-induced increase in expression of tumor necrosis factor alpha (TNF-α) in the rat hippocampus parallel with its anti-seizure effect [39]. Repeated application of levetiracetam (orally, 360 mg/kg for 2 days) attenuated the expression of interleukin-1β (IL-1β) and TNF-α in the hippocampus of mice after status epilepticus [40]. Moreover, levetiracetam (i.p. 30 mg/kg for 1 week) reduced the IL-1β and interleukin-1 receptor subtype 1 (IL-1R1) expression in astrocytes and microglia as well as the reactive gliosis in the piriform cortex and hippocampus of epileptic rats [41]. Oral administration of levetiracetam (180 and 360 mg/kg for 1–7 days) prevented upregulation of expression of pro-inflammatory molecules and related receptors, such as IL-1β, TNFα, inducible nitric oxide synthase (iNOS), IL-1R1 and Toll-like receptor 4 (TLR4), in the hippocampus in post-status epilepticus mice [42]. The administration of therapeutic levetiracetam doses decreased serum IL-1β level in patients with different types of epilepsies [43] as well as serum IL-1β and TNF-α level in healthy female subjects [44]. Moreover, IL-1β can attenuate the GABAergic transmission and enhance excitatory glutamatergic neurotransmission [45,46], whereas TNF-α can mitigate GABA_A_R-induced inhibition and increase AMPA-dependent excitation [47,48]. It has been demonstrated that both IL-1β and TNF-α inhibit astrocyte glutamate uptake, resulting in an enhanced extracellular glutamate level [49,50]. All of these inflammatory effects above may generate (or enhance) an imbalance between excitatory and inhibitory processes, resulting in excessive excitation in brain areas, such as the cortex [51], therefore increasing the possibility of the appearance (or aggravation) of epileptic seizures. Consequently, levetiracetam may be able to mitigate or abolish epileptic activity through inhibition of inflammatory processes-evoked pro-epileptic influences.

It was also demonstrated that levetiracetam was not able to induce significant histone hyperacetylation directly in the human cervical carcinoma cell line HeLa. However, levetiracetam metabolite 2-pyrrolidinone-n-butyric acid (PBA) can inhibit the transcription repressor histone deacetylases (HDACs), therefore inducing the accumulation of acetylated histones [52]. These results suggest that levetiracetam treatment is also able to modulate gene expression through histone hyperacetylation enhancement indirectly [52]. Consequently, levetiracetam can promote the transcription of several genes resulting in neuroprotective influences, such as anti-inflammatory effects [53], and protection against mitochondrial dysfunction in experimental status epilepticus in rats [54].

In relation to the mechanism of action of levetiracetam on SWDs in WAG/Rij rats, it was demonstrated, among others, that enhanced expression of the *P*/*Q*-type calcium channel could be related to the occurrence of SWDs [55], suggesting that levetiracetam treatment may mitigate SWDs through inhibition of this calcium channel in WAG/Rij rats. Moreover, both increase in the activity of the GABAergic system and decrease in the activity of the glutamatergic system may result in decrease in the SWD number and SWD time by reduction in hyperexcitability in the somatosensory cortex (cortical focus of absence epilepsy genesis) [2,56,57]. Consequently, it was suggested that levetiracetam can evoke anti-absence epileptic activity through the GABAergic mechanism, likely by increasing the GABA level in WAG/Rij rats [16]. However, thalamic increase in GABAergic inhibition can generate/enhance SWDs in WAG/Rij rats [56]. Thus, these results suggest that levetiracetam application can exert a beneficial effect on absence epileptic activity not only through decrease in the glutamate level in brain areas implicated in absence epilepsy genesis, but also by treatment-evoked decrease (in the thalamus) and increase (in the cortex) in GABA levels in WAG/Rij rats. Pro-inflammatory cytokines, such as IL-1β and TNF-α, as well as lipopolysaccharide (LPS)-evoked increase in their levels, can increase the SWD number and SWD time in WAG/Rij rats [58,59,60,61]. Consequently, levetiracetam treatment can also decrease SWDs through neuroprotective, anti-inflammatory effects. It was demonstrated that adenosine can (i) mitigate neuroinflammation mainly via A_1_Rs; (ii) hyperpolarize neuronal membrane potential and decrease neuronal hyperexcitability through, among others, A_1_R-evoked enhancement of ATP-sensitive potassium (K_ATP_) channel activity; and (iii) decrease function of high-voltage calcium channels, such as *P*/*Q*-type channels [62,63,64,65]. All these influences of enhanced A_1_R activity, evoked by levetiracetam treatment, can mitigate SWDs in WAG/Rij rats.

**Figure 7:**
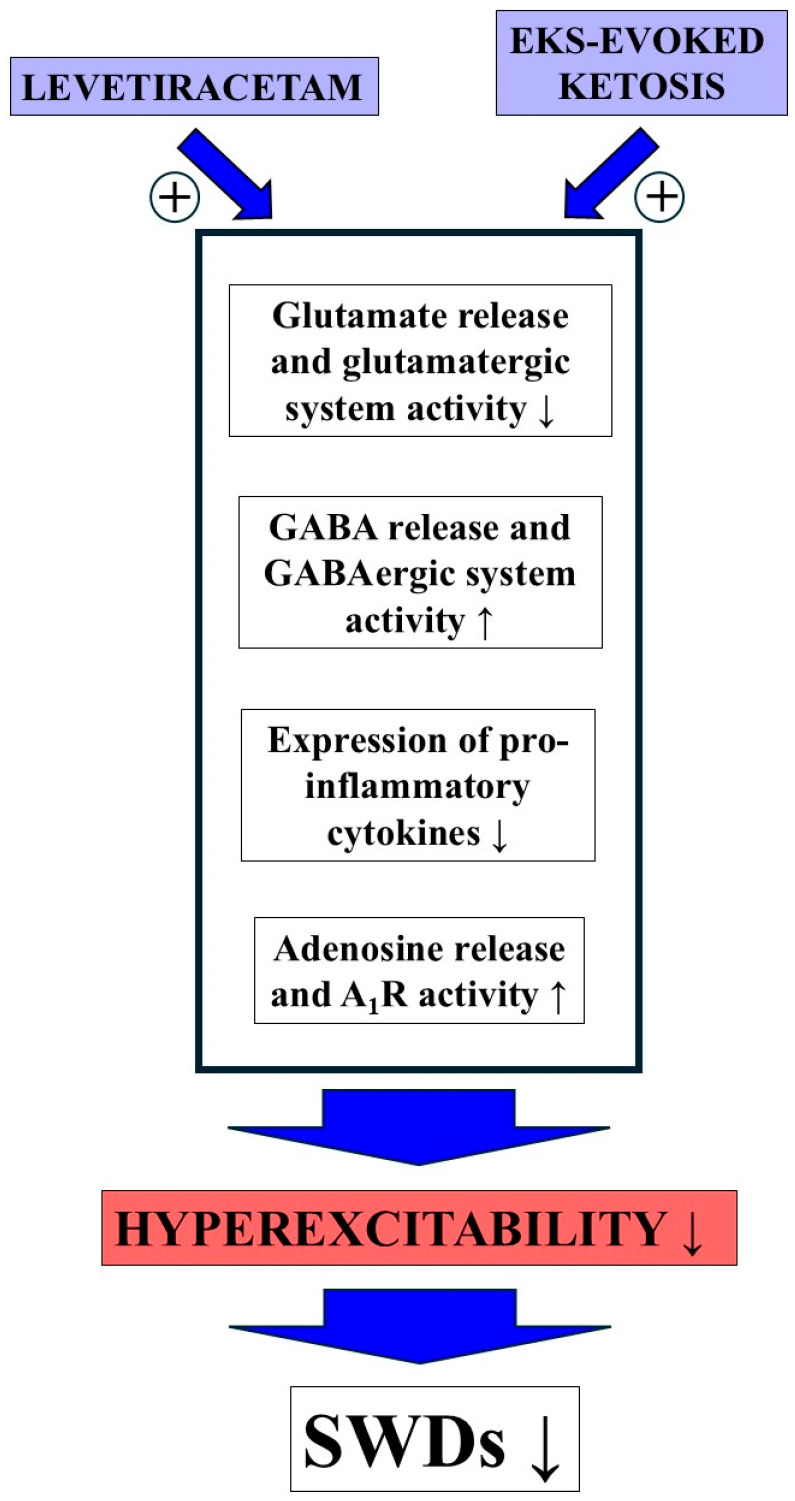
Main putative common mechanisms of action of levetiracetam and EKS (e.g., KEKS) treatment leading to an enhanced anti-absence epileptic effect of combined administration (e.g., decrease in SWD number). Abbreviations: A_1_R, adenosine A1 receptor; GABA, gamma-amino butyric acid; EKS, exogenous ketone supplement; SWD, spike-wave discharge.

EKSs are known to increase blood ketone body levels [8,9], by which they are able to evoke beneficial effects on several types of central nervous system diseases, such as epilepsy in both animals and human patients [9,11]. In relation to the putative mechanisms of action of EKS-evoked ketosis on absence epilepsy, studies show that ketosis can (i) decrease glutamate and increase GABA level in the brain and therefore hyperpolarize the neuronal membrane [66,67]; (ii) increase both adenosine level and A_1_R activity, by which it may increase activity of the K_ATP_ channel, therefore decreasing membrane potential and hyperexcitability, and mitigating inflammatory processes, as well as oxidative stress [13,68,69]; (iii) function as an HDAC inhibitor leading to, among others, decrease in pro-inflammatory cytokine level and oxidative stress [68]; and (iv) decrease inflammatory processes (e.g., mitigate IL-1β level) and oxidative stress (e.g., decrease in iNOS activity) through hydroxycarboxylic acid receptor 2 (HCAR2) [70]. These effects, as was suggested above, could evoke a decrease in absence epileptic activity in WAG/Rij rats. Indeed, EKSs, such as KE and KEKS, as well as KSMCT effectively decreased both the spontaneously developed SWDs and LPS-evoked increase in SWD number in WAG/Rij rats [9,19], likely through a partly restored balance between excitatory and inhibitory processes in the brain. Based on these results, the EKSs-evoked anti-absence epileptic mechanism of action (and its pathways) may be similar, at least partly, to the levetiracetam-generated multiple influences, such as modulation of function of the GABAergic, glutamatergic and adenosinergic systems, as well as neuroprotective, anti-inflammatory and epigenetic influences, leading to integration of different pharmacological influences and therefore an enhanced anti-absence epileptic effect (Figure 7).

The main limitations of the current study are that we used only (i) one dose for both EKSs in KEKS food (10% KE + 10% KS) and levetiracetam (i.p 200 mg/kg) and (ii) 5 days treatment (iii) on 10-months-old male WAG/Rij rats to investigate the modulatory influence of EKSs on the levetiracetam-evoked effect on SWDs. Moreover, the current study did not investigate the treatment-generated changes in neurotransmitter systems, such as alteration in neuromodulator and neurotransmitter (e.g., glutamate, GABA and adenosine) levels and signaling processes. Consequently, although the current study extended our previous results on the effects of ketone supplementation on absence epileptic activity on similarly aged male WAG/Rij rats [9,19,20,24], additional studies are needed using (i) other doses of EKSs and levetiracetam to reveal the dose-dependent influence of their combined chronic treatments on SWDs; (ii) testing of the effects of long-term administration; (iii) other animal models of human absence epilepsy, such as Genetic Absence Epilepsy Rats from Strasbourg (GAERS) [71] to disclose or exclude rat strain-dependent effects of the treatments; (iv) not only male, but also female animals to investigate the putative sex-dependent effects of treatments; and (v) different additional analytical techniques, such as mass spectrometry, to explore both the putative changes in neurotransmitter systems and the exact mechanisms of action of the combined treatment method. These future studies could further confirm the presented results and could help us to understand the treatment-enhancing effect of ketone supplementation on the levetiracetam-generated anti-epileptic influence. Moreover, in relation to blood glucose levels, the identified low statistical power (0.06) suggests that the study was underpowered to detect small differences in glucose levels, and thus nonsignificant findings for this measure should be interpreted with caution. Thus, future studies with larger sample sizes are warranted to confirm these results.

## 5. Conclusions

In this study, it was demonstrated that combined administration of a ketone supplement (KEKS food) with levetiracetam can enhance the anti-absence epileptic influence of levetiracetam in WAG/Rij rats. Consequently, we can propose that combined application of proper doses of levetiracetam and EKSs could significantly improve the therapeutic effect of levetiracetam in patients with absence epilepsy. Moreover, theoretically, the combination of levetiracetam with EKSs may reduce the dose of levetiracetam required in therapy. Thus, based on our results, we can suggest that application of levetiracetam with EKSs for the treatment of absence epilepsy, especially in patients with refractory, drug-resistant seizures, can be recommended. Furthermore, it can be hypothesized that combined administration using not only levetiracetam, but also other anti-epileptic drugs with EKSs may represent a potential approach to the clinical treatment of patients with not only absence epilepsy, but also other types of epilepsies. However, to support our conclusion, future mechanistic studies are needed, as are dose-optimization studies, of both levetiracetam and EKSs alone and in combination on epileptic activity in both animal models and human patients.

## Figures and Tables

**Table 1:** Effect of levetiracetam treatment alone (i.p. 200 mg/kg) on SWDs.

Treatments	Group 1 (Figure 2)					
	Mean ± S.E.M. (Significance Level/*p* Value)					
	SWD Number		SWD Duration (s)		Time Spent in SWD (s)	
	30–90 min	90–150 min	30–90 min	90–150 min	30–90 min	90–150 min
Control (CON; i.p. saline + normal rat food)	24.2 ± 2.99	20.1 ± 2.37	4.2 ± 0.30	4.2 ± 0.23	105.6 ± 18.75	82.5 ± 10.80
1st treatment day (1D; i.p. 200 mg/kg levetiracetam + normal rat food)	6.1 ± 0.89 ****/<0.0001	5.1 ± 1.47 ****/<0.0001	3.1 ± 0.18 */0.0486	3.1 ± 0.24 ns/0.0586	19.4 ± 3.35 ****/<0.0001	15.6 ± 4.31 ****/<0.0001
2nd treatment day (2D; i.p. 200 mg/kg levetiracetam + normal rat food)	10.0 ± 1.79 ****/<0.0001	4.7 ± 2.16 ****/<0.0001	3.00 ± 0.29 */0.016	3.1 ± 0.33 ns/0.1178	31.4 ± 7.82 ****/<0.0001	14.7 ± 6.68 ****/<0.0001
3rd treatment day (3D; i.p. 200 mg/kg levetiracetam + normal rat food)	9.9 ± 2.13 ****/<0.0001	7.6 ± 3.17 ***/0.0007	3.1 ± 0.20 ns/0.0769	3.16 ± 0.33 ns/0.1497	31.5 ± 7.21 ****/<0.0001	22.5 ± 8.65 ****/<0.0001
4th treatment day (4D; i.p. 200 mg/kg levetiracetam + normal rat food)	9.7 ± 1.81 ****/<0.0001	8.6 ± 2.00 **/0.0024	3.1 ± 0.28 ns/0.0586	2.9 ± 0.20 */0.033	33.2 ± 8.07 ****/<0.0001	25.7 ± 6.24 ***/0.0002
5th treatment day (5D; i.p. 200 mg/kg levetiracetam + normal rat food)	8.1 ± 1.55 ****/<0.0001	8.1 ± 0.94 **/0.0015	3.4 ± 0.43 ns/0.2658	3.0 ± 0.16 ns/0.0534	30.0 ± 7.06 ****/<0.0001	24.8 ± 3.36 ***/0.0002

Abbreviations: 1D, first treatment day; 2D, second treatment day and so on; CON, control; normal rat food, paste-like normal rat food (powdered standard rodent chow, 1% saccharine and water); ns, non-significant; SWD, spike-wave discharge; level of significance: *, *p* < 0.05; **, *p* < 0.01; ***, *p* < 0.001; ****, *p* < 0.0001.

**Table 2:** Effect of KEKS food alone on SWDs.

Treatments	Group 2 (Figure 3)					
	Mean ± S.E.M. (Significance Level/*p* Value)					
	SWD Number		SWD Duration (sec)		Time Spent in SWD (sec)	
	30–90 min	90–150 min	30–90 min	90–150 min	30–90 min	90–150 min
Control (CON; i.p. saline + normal rat food)	23.7 ± 4.87	20.4 ± 2.22	5.3 ± 0.27	5.1 ± 0.28	130.3 ± 30.26	106.1 ± 14.84
1st treatment day (1D; i.p. saline + KEKS food)	18.4 ± 2.44 ns/0.8451	18.0 ± 3.37 ns/0.9939	4.1 ± 0.20 ns/0.0715	4.0 ± 0.25 ns/0.1133	74.3 ± 10.88 ns/0.1454	72.1 ± 13.67 ns/0.664
2nd treatment day (2D; i.p. saline + KEKS food)	17.0 ± 4.82 ns/0.6641	12.7 ± 2.82 ns/0.5102	3.9 ± 0.41 */0.0255	3.6 ± 0.41 */0.0146	72.3 ± 27.74 ns/0.1199	49.7 ± 14.58 ns/0.1398
3rd treatment day (3D; i.p. saline + KEKS food)	15.9 ± 4.78 ns/0.4978	15.7 ± 2.19 ns/0.894	2.8 ± 0.49 ****/<0.0001	3.5 ± 0.21 **/0.0081	52.2 ± 16.05 */0.0115	57.4 ± 10.29 ns/0.274
4th treatment day (4D; i.p. saline + KEKS food)	8.4 ± 2.28 */0.0396	10.4 ± 0.61 ns/0.2257	4.0 ± 0.33 ns/0.0608	3.9 ± 0.23 ns/0.0976	44.5 ± 15.11 **/0.004	41.0 ± 3.12 ns/0.0568
5th treatment day (5D; i.p. saline + KEKS food)	7.3 ± 1.06 **/0.0055	9.1 ± 1.18 ns/0.1246	4.0 ± 0.29 */0.0435	3.5 ± 0.18 **/0.0081	27.9 ± 3.74 ***/0.0003	33.1 ± 5.54 */0.0225

Abbreviations: 1D, first treatment day; 2D, second treatment day and so on; CON, control; ns, non-significant; SWD, spike-wave discharge; level of significance: *, *p* < 0.05; **, *p* < 0.01; ***, *p* < 0.001; ****, *p* < 0.0001.

**Table 3:** Influence of combined administration of levetiracetam (i.p. 200 mg/kg) with KEKS food on SWDs.

Treatments	Group 3 (Figure 4)					
	Mean ± S.E.M. (Significance Level/*p* Value)					
	SWD Number		SWD Duration (sec)		Time Spent in SWD (sec)	
	30–90 min	90–150 min	30–90 min	90–150 min	30–90 min	90–150 min
Control (CON; i.p. saline + normal rat food)	24.7 ± 4.55	18.8 ± 1.83	4.8 ± 0.25	4.7 ± 0.21	120.3 ± 23.80	91.0 ± 10.91
1st treatment day (1D; i.p. 200 mg/kg levetiracetam + KEKS food)	4.9 ± 0.96 ****/<0.0001	5.6 ± 1.38 ****/<0.0001	3.2 ± 0.28 ns/0.0752	3.2 ± 0.16 ns/0.0707	15.9 ± 3.30 ****/<0.0001	18.5 ± 5.15 ****/<0.0001
2nd treatment day (2D; i.p. 200 mg/kg levetiracetam + KEKS food)	3.9 ± 0.80 ****/<0.0001	3.6 ± 0.84 ****/<0.0001	3.3 ± 0.18 ns/0.1071	3.1 ± 0.41 ns/0.0516	13.4 ± 3.15 ****/<0.0001	10.7 ± 2.54 ****/<0.0001
3rd treatment day (3D; i.p. 200 mg/kg levetiracetam + KEKS food)	4.3 ± 0.78 ****/<0.0001	2.6 ± 0.69 ****/<0.0001	3.1 ± 0.14 */0.0484	2.5 ± 0.46 **/0.0024	13.3 ± 2.49 ****/<0.0001	7.4 ± 2.03 ****/<0.0001
4th treatment day (4D; i.p. 200 mg/kg levetiracetam + KEKS food)	2.7 ± 1.17 ****/<0.0001	4.0 ± 0.72 ****/<0.0001	2.4 ± 0.89 **/0.0013	2.7 ± 0.11 **/0.0075	8.1 ± 3.07 ****/<0.0001	11.0 ± 2.11 ****/<0.0001
5th treatment day (5D; i.p. 200 mg/kg levetiracetam + KEKS food)	2.9 ± 0.71 ****/<0.0001	1.7 ± 0.52 ****/<0.0001	2.4 ± 0.41 ***/0.0009	2.6 ± 0.57 **/0.0047	8.2 ± 2.41 ****/<0.0001	5.1 ± 1.50 ****/<0.0001

Abbreviations: 1D, first treatment day; 2D, second treatment day and so on; CON, control; ns, non-significant; SWD, spike-wave discharge; level of significance: *, *p* < 0.05; **, *p* < 0.01; ***, *p* < 0.001; ****, *p* < 0.0001.

**Table 4:** Effect of KEKS food alone and combined administration of levetiracetam (i.p. 200 mg/kg) with KEKS food on blood R-βHB and glucose levels.

Treatments	Mean ± S.E.M. (Significance Level/*p* Value)	
	R-βHB (mmol/L)	Glucose (mg/dL)
Group 2 (Figure 6A,B)		
Last control day (CON; i.p. saline + normal rat food)	0.8 ± 0.03	74.0 ± 2.17
1st treatment day (1D; i.p. saline + KEKS food)	1.2 ± 0.05 ***/0.0005	69.3 ± 2.76 ns/0.4284
5th treatment day (5D; i.p. saline + KEKS food)	1.2 ± 0.04 ****/<0.0001	72.3 ± 3.21 ns/0.5219
Group 3 (Figure 6C,D)		
Last control day (CON; i.p. saline + normal rat food)	0.8 ± 0.04	75.1 ± 1.89
1st treatment day (1D; i.p. 200 mg/kg levetiracetam + KEKS food)	1.2 ± 0.04 ***/0.0002	68.3 ± 2.20 ns/0.0523
5th treatment day (5D; i.p. 200 mg/kg levetiracetam + KEKS food)	1.3 ± 0.04 ***/0.0003	73.0 ± 2.15 ns/0.6092

Abbreviations: 1D, first treatment day; 5D, fifth treatment day; CON, control; R-βHB, R-beta-hydroxybutyrate; ns, non-significant; level of significance: ***, *p* < 0.001; ****, *p* < 0.0001.

## Data Availability

The original contributions presented in this study are included in the article. Further inquiries can be directed to the corresponding author.

## Abbreviations

A_1_R, adenosine A1 receptor; AMPA, alpha-amino-3-hydroxy-5-methylisoxazole-4-propionic acid; ATP, adenosine triphosphate; βHB, beta-hydroxybutyrate; EEG, electroencephalogram; EKS, exogenous ketone supplement; ENT1, equilibrative nucleoside transporter; GABA, gamma-amino butyric acid; GABA_A_R, GABA_A_ receptor; HDAC, histone deacetylase; IL-1β, interleukin-1β; IL-1R1, interleukin-1 receptor subtype 1; iNOS, inducible nitric oxide synthase; i.p., intraperitoneal; K_ATP_ channel, ATP-sensitive potassium channel; KE, ketone ester; KEKS, mix of KE and KS; Kir3.2, potassium inwardly-rectifying channel 3.2; KS, ketone salt; KSMCT, a mix of KS and MCT oil; LPS, lipopolysaccharide; MCT, medium-chain triglyceride; SV2A, synaptic vesicle protein 2A; SWD, spike-wave discharge; TNF-α, tumor necrosis factor alpha; WAG/Rij, Wistar Albino Glaxo/Rijswijk.
